# A Novel Ourmia-Like Mycovirus Confers Hypovirulence-Associated Traits on *Fusarium oxysporum*

**DOI:** 10.3389/fmicb.2020.569869

**Published:** 2020-12-09

**Authors:** Ying Zhao, Yuanyan Zhang, Xinru Wan, Yuanyuan She, Min Li, Huijun Xi, Jiatao Xie, Caiyi Wen

**Affiliations:** ^1^College of Plant Protection, Henan Agricultural University, Zhengzhou, China; ^2^State Key Laboratory of Agricultural Microbiology, Huazhong Agricultural University, Wuhan, China

**Keywords:** *Fusarium oxysporum*, ourmia-like virus, hypovirulence, mycovirus, transfection

## Abstract

Fusarium wilt caused by *Fusarium oxysporum* f. sp. *momordicae* (*FoM*) is an important fungal disease that affects the production of bitter gourd. Hypovirulence-associated mycoviruses have great potential and application prospects for controlling the fungal disease. In this study, a novel ourmia-like virus, named *Fusarium oxysporum* ourmia-like virus 1 (FoOuLV1), was isolated from *FoM* strain HuN8. The viral genomic RNA is 2,712 nucleotides (nt) in length and contains an open reading frame (ORF) encoding a putative RNA-dependent RNA polymerase (RdRp) using either standard or mitochondrial codes. In strain HuN8, there was also a FoOuLV1-associated RNA segment with 1,173 nt in length with no sequence homology. Phylogenetic analysis showed that FoOuLV1 is a member of the genus *Magoulivirus* of the family *Botourmiaviridae*. FoOuLV1 was found to be associated with hypovirulence in *FoM*. Moreover, FoOuLV1 and its hypovirulence trait can be transmitted horizontally to other *FoM* strains and also to other formae speciale strains of *F. oxysporum*. In addition, FoOuLV1 showed significant biological control effect against the bitter gourd Fusarium wilt. To our knowledge, this study reveals the first description of a hypovirulence-associated ourmia-like mycovirus, which has the potential to the biological control of Fusarium wilt.

## Introduction

Mycoviruses are widespread in all major filamentous fungi, yeasts, and oomycetes (Xie and Jiang, 2014; Ghabrial et al., 2015). According to a recent study, more than 300 mycoviral sequences have been logged in the National Center for Biotechnology Information (NCBI) database (Zhou et al., 2020), which are grouped into at least 19 families by the International Committee on Taxonomy of Viruses (ICTV). Most mycoviruses have double-stranded RNA (dsRNA); however, some mycoviruses have positive single-stranded RNA (+ssRNA), and a few mycoviruses have single-stranded DNA (ssDNA) or negative single-stranded RNA (−ssRNA; Ghabrial and Suzuki, 2009; Xie and Jiang, 2014; Ghabrial et al., 2015). The ssRNA mycoviruses are grouped into seven families: *Alphaflexiviridae*, *Barnaviridae*, *Botourmiaviridae*, *Gammaflexiviridae*, *Hypoviridae*, *Mymonaviridae*, and *Narnaviridae* (Amarasinghe et al., 2018). There are also unclassified mycoviruses (Li H. et al., 2019).

*Ourmiavirus* is a genus of viruses isolated from various plants. The genome of *Ourmiavirus* contains three linear + ssRNAs, each encoding a single protein: RNA-dependent RNA polymerase (RdRp), movement protein (MP), or capsid protein (CP; Lisa et al., 1988; Turina et al., 2017). The *Ourmiavirus* has a unique bacilliform virion structure (Avgelis et al., 1989). Viruses in the family *Narnaviridae* have a 2.5- to 3.0-kb genome that encodes only an RdRp with no capsid; they have been widely discovered in fungi, oomycetes, invertebrates, and plants (Cai et al., 2012; Bruenn et al., 2015; Shi et al., 2016). Recently, many mycoviruses containing only one RNA segment encoding RdRp have been identified to be phylogenetically related to *Ourmiavirus* genus (Xu et al., 2015; Shi et al., 2016; Amarasinghe et al., 2018; Zheng et al., 2019; Li C.X. et al., 2019). Therefore, the ICTV created a new family (*Botourmiaviridae*) to incorporate these ourmia-like viruses (Turina et al., 2017; Wang et al., 2020). *Botourmiaviridae* comprising five genera: *Magoulivirus*, *Ourmiavirus*, *Botoulivirus*, *Scleroulivirus*, and *Penoulivirus* (Zhou et al., 2020). Whether these ourmia-like mycoviruses have other RNA segments remains unknown (Wang et al., 2020).

The mycoviruses infection usually causes no associated symptoms and sometimes even has beneficial effects on their fungal hosts (Zheng et al., 2019). Some mycoviruses in plant pathogenic fungi can reduce the ability of their fungal hosts to cause disease in plants. This property, named hypovirulence, has explored for biological control of crop fungal diseases. (Nuss, 2005; Xie and Jiang, 2014). Cryphonectria hypovirus 1 (CHV1) and Sclerotiorum sclerotiorum hypovirulence-associated DNA virus 1 (SsHADV-1) have been successfully used to control diseases caused by *Cryphonectria parasitica* and *Sclerotiorum sclerotiorum* (Anagnostakis, 1982; Yu et al., 2013). Besides these examples, many hypovirulence-associated mycoviruses, belonging to various genera, have been identified from diverse plant pathogenic fungi, such as Hubei sclerotinia RNA virus 1 (HuSRV1), Botryosphaeria dothidea botourmiavirus 1 (BdBOV-1), Alternaria alternata hypovirus 1 (AaHV1), and Rhizoctonia solani endornavirus 1 (RsEV1), etc. (Xie et al., 2011; Azhar et al., 2019; Zheng et al., 2019; Li H. et al., 2019). However, to our knowledge, no hypovirulence-associated ourmia-like mycovirus has been reported so far.

Fusarium is widely distributed in soil, associated with plants worldwide. It includes many important plant pathogenic fungi (Li P. et al., 2019). Many mycoviruses have been reported in different species of *Fusarium* genus, and a few of them have hypovirulent effects on their hosts, including: Fusarium graminearum virus 1 (FgV1), Fusarium graminearum virus-ch9 (FgV-ch9), Fusarium graminearum hypovirus 2 (FgHV2), and so on (Kwon et al., 2007; Darissa et al., 2012; Li et al., 2015). *F. oxysporum* is an important plant pathogenic fungus and causes vascular wilt in a wide variety of agricultural crop species. *F. oxysporum* is classified into different host-specific forms (formae speciales) based on the types of host plants (Di Pietro et al., 2003). Although a number of mycoviruses have been identified in the genus *Fusarium*, only three mycoviruses have been reported in *F. oxysporum*. *Fusarium oxysporum* chrysovirus 1 (FoCV1) was found in *F. oxysporum* f. sp. *melonis* and assigned to the family *Chrysoviridae*, but its complete genomic sequence has not been determined (Sharzei et al., 2007). *Fusarium oxysporum* f. sp. dianthi mycovirus 1 (FodV1), a new member of the family *Chrysoviridae*, has been isolated from *F. oxysporum* f. sp. *dianthi* and exerts a hypovirulent effect (Lemus-Minor et al., 2015). In addition, *Fusarium oxysporum* f. sp. dianthi mitovirus 1 (FodMV1) has been identified from *F. oxysporum* f. sp. *dianthi*, but it has no hypovirulence trait (Torres-Trenas and Pérez-Artés, 2020).

In this study, we identified and characterized a novel hypovirulence-inducing ourmia-like mycovirus from *F. oxysporum* f. sp. *momordicae* named *Fusarium oxysporum* ourmia-like virus 1 (FoOuLV1) and verified its hypovirulence trait and its horizontal transmission ability. In addition, we evaluated the biocontrol potential of FoOuLV1 by the pot and field experiments.

## Materials and Methods

### Fungal Isolates and Plant Materials

*Fusarium oxysporum* f. sp. *momordicae* strains HuN8 and SD-1 were isolated from bitter gourd, showing symptoms of Fusarium wilt disease in Hunan and Shandong Province, China, in 2017. *F. oxysporum* f. sp. *cucumerinum* (*FoC*) strain HK3 was kindly gifted by Dr. Xuehong Wu (China Agricultural University, Beijing, China). The strains were cultured on potato dextrose agar (PDA) medium at 28°C. Fungal DNA was isolated using standard phenol-chloroform extraction and ethanol precipitation, then used for polymerase chain reaction (PCR) amplification with universal primers (ITS1 and ITS4) and specific primers (fp7318 and fp7335) to perform formae speciales identification of *F. oxysporum* (van Dam et al., 2018). Bitter gourd seeds were purchased from Fujian Agricultural Science Agricultural Seed Development Co. Ltd. All primers used in this manuscript are listed in Supplementary Table 1.

### RNA Extraction and RT-PCR Detection

The extraction of ssRNA and dsRNA was performed following the procedure described previously (Xie et al., 2006). Strains grew for 4–5 days on cellophane membranes of the PDA medium. Fresh mycelia (1–2 *g*) were harvested to isolate dsRNA. 2 × GPS (glycine 15.0 g/L, Na_2_HPO_4_ 14.2 g/L, NaCl 35.1 g/L, and pH = 9.6) 400 μl, phenol (pH 8.0) 400 μl, chloroform-isoamyl alcohol (24:1) 400 μl, and 10% SDS 92 μl were added to a sterile centrifuge tube each 0.2 *g* sample and were shaken for 10 min at room temperature, then centrifuged at 12,000 revolutions per minute (rpm) for 10 min. The supernatant was transferred to another clean centrifuge tube and mixed with 114 μl anhydrous ethanol for every 600 μl of the supernatant. Then 0.04 *g* cellulose powder CF-11 (Sigma–Aldrich, St. Louis, MO, United States) was added. The supernatant was shaken for 30 min in an ice bath. After centrifugation at 12,000 rpm for 1 min, the supernatant was discarded, 600 μl of eluent buffer [10 × STE (0.5 M Tris; 1 M NaCl, 10 mM EDTA, and pH = 7.0) 10 ml, 95% ethanol 17 ml, DEPC-H_2_O 73 ml] was added to elute for two to three times. After centrifugation at 12,000 rpm for 2 min, the supernatant was discarded and added with 600 μl 1 × STE, mixed and shaken for 5 min, and centrifuged at 12,000 rpm for 5 min. Then the supernatant was transferred to a new centrifuge tube, and the same volume isopropyl alcohol, with acryl carrier nucleic acid co-precipitator (item No. RP2001, ^®^Bioteke Corporation, Beijing, China) were added to improve the yield of dsRNA. After centrifugation at 12,000 rpm at 4°C for 25 min, the precipitation was washed with 75% ethanol, dried at 37°C for 10 min, and dissolved in 25 μl DEPC-H_2_O. The dsRNA was treated with RNase-free DNase I (0.6 U/μl) and S1 nuclease (3 U/μl). The segments were electrophoresed on a 1% agarose gel, stained with ethidium bromide, and visualized with gel documentation and image analysis system (InGenius LhR, Syngene, United Kingdom).

To extract total RNA, strains were grown on the cellophane membrane overlying a PDA plate for 4–5 days, and 100–200 mg fresh mycelia were harvested and ground to powder in liquid nitrogen. Total RNA was prepared using an RNA reagent (Newbio Industry, Wuhan, China), according to the manufacturer’s instructions.

For RT-PCR detection, first-strand cDNAs were synthesized using TransScript One-Step gDNA Removal and cDNA Synthesis SuperMix (^®^TransGen Biotech, Beijing, China) according to the manufacturer’s instructions. Then the PCR amplification was carried out using the specific primers (FV-L-S and FV-L-A for L-segment; FV-S-S and FV-S-A for S-segment). All primers are listed in Supplementary Table 1.

### cDNA Cloning and Sequencing

The cDNA library was constructed using TransScript One-Step gDNA Removal and cDNA Synthesis SuperMix (TransGen Biotech) according to the manufacturer’s instructions.

To obtain initial sequence clones, a random primer (RACE3RT) was used for RT-PCR amplification. The PCR products were cloned into a pMD18-T vector (Takara, Dalian, China) for Sanger sequencing, with the internal gaps between the initial sequences filled by RT-PCR. The terminal sequence cloning was performed according to the method previously described (Potgieter et al., 2009). All primers used for cDNA cloning and sequencing are listed in Supplementary Table 1.

### Sequence and Phylogenetic Analysis

Open reading frames (ORFs) and conserved domains were predicted using ORF finder and CD-search on the website of NCBI^1^ and motifs scan website^2^. Multiple sequence alignments were performed, and the phylogenetic tree was constructed using MEGA7 software (Kumar et al., 2016). Potential secondary structures were predicted by Mfold version 2.3 (Zuker, 2003).

### Viral Transmission Assay

Hyphal fusion was conducted to investigate viral horizontal transmission between *Fusarium oxysporum* f. sp. *momordicae* (*FoM*) strains and *FoC* strains, following the procedure described previously (Li H. et al., 2019). The SD-1Hyg^*R*^ and HK3 Hyg^*R*^ strains which obtained hygromycin resistance by agrobacterium-mediated transformation from the strains SD-1 and HK3. The agrobacterium-mediated transformation was following the procedure described previously (Mullins et al., 2001). The agrobacterium (EHA105) and the plasmid (pTFCM) were kindly gifted by Dr. Daohong Jiang (Huazhong Agricultural University, Wuhan, China), which were described previously (Li et al., 2005). The biological phenotype of strains SD-1Hyg^*R*^ and HK3 Hyg^*R*^ were the same as the strains SD-1 and HK3.

A schematic diagram of the co-culturing performed using the viral transmission assay is shown in Supplementary Figure 1. Strains HuN8, SD-1HygR, and HK3Hyg^*R*^ were grown for 4–5 days on PDA medium. Then, mycelia blocks of strains HuN8 and the SD-1Hyg^*R*^ (or HK3Hyg^*R*^) were inoculated on the same PDA plate, growing for 4–5 days until the colony of HuN8 and the SD-1HygR (or HK3HygR) covered each other. Mycelia blocks of the common colony were transferred to new PDA plates with hygromycin (50 mg/ml), growing for 2–3 days. The mycelia could grow on the hygromycin-resistant PDA plates and became the derivative strains. The viral transmission was evaluated based on dsRNA extraction or RT-PCR detection.

### Virulence Assay of *in vitro* Inoculation and Pot and Field Experiments

The virulence of *FoM* strains was assessed by *in vitro* inoculation and the pot and field experiments on bitter gourd plants. For *in vitro* experiment, young leaves and petioles of bitter gourd were inoculated with *FoM* strains, moisturized for 4–5 days, observed, and photographed. The leaves inoculated with nothing were used as control. Four leaves were used for a group in each experiment, and all the experiments were repeated three times.

For the pot experiment, bitter gourd seedlings with two or three leaves grown in sterile soil were inoculated with 10 ml of *F. oxysporum* spore (10^7^ ml^–1^) suspension into the root, cultivated in the growth chamber, observed, and photographed. The plants inoculated with water were used as control. Four plants were used for a group in each experiment, and all the experiments were repeated three times.

The virulence assay of *FoC* strains was similar with the *FoM* strains. Cucumber seedlings with two leaves grown in sterile soil were inoculated with 10 ml of *F. oxysporum* spores (10^7^ ml^–1^) suspension, cultivated, observed, and photographed. Plants inoculated with water were used as control. Four plants were used for a group in each experiment, and all the experiments were repeated three times.

The field experiment was conducted from 21 May 2019 to 16 July 2019 in two fields where Fusarium wilt had been present for many years in Yuanyang, Henan Province, China. The bitter gourd seedlings (about 30 seedlings for each group in each field) were planted according to the experimental design (a schematic representation of the experiment distribution is shown in Supplementary Figure 2). In the treatment group, 10 ml of spores (10^7^ ml^–1^) of the SD-V strains were inoculated into the area surrounding the roots on 21 May 2019; at the same time, the control group was instead inoculated with water. The disease incidence, severity, and index were assessed twice, on 25 June and on 16 July. Disease incidence was defined as the percentage of infected plants, and disease severity was rated on a scale of 0–9 as follows: level 0, no symptoms; level 1, <30% of leaves showing leaf veins with yellowing; level 3, 30–70% of leaves showing leaf veins with yellowing; level 5, leaf veins on the whole plant are yellowed, but the growth and development of the plant are not affected; level 7, the whole plant is yellowed and wilted, and the vascular bundle has turned brown and stopped growing and developing; and level 9, the plant is dead. The different levels of symptoms are shown in Supplementary Figure 3.

## Results

### Viruses in *F. oxysporum* f. sp. *momordicae* Strain HuN8

Total RNA was extracted from the *FoM* strain to perform NGS. The assembled sequences were used for homology searches against the NCBI virus amino acid sequence database using BLASTX. Eight contigs could be assembled into one long contig from the database. The long contig was similar to the sequence of Magnaporthe oryzae ourmia-like virus (SBQ28480.1), thus was named FoOuLV1.

**FIGURE 1:**
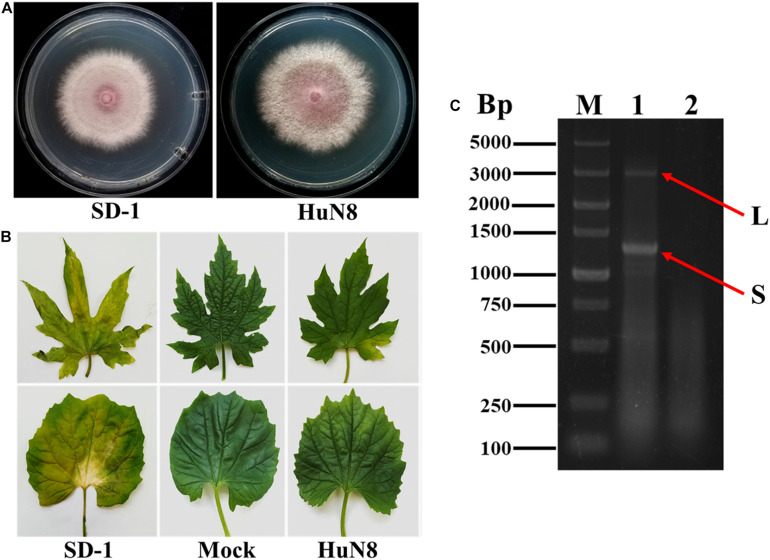
The biological characteristics and dsRNA pattern of *Fusarium oxysporum* f. sp. *momordicae* strain HuN8. **(A)** Colony morphology of strain HuN8 and virulent strain SD-1 (cultured on PDA for 2 days at 28°C). **(B)** Pathogenicity of strain HuN8 and SD-1 on the detached bitter gourd leaves (72 h post-inoculation at 28°C). Mock, blank control. **(C)** Agarose gel electrophoretic analysis of the dsRNA extracted from strain HuN8. The dsRNA was treated with DNase I. M, molecular weight marker; 1, dsRNA extracted from strain HuN8; and 2, dsRNA extracted from strain SD-1.

Based on the RT-PCR analysis, we found that FoOuLV1 was harbored in *FoM* strain HuN8, which was isolated from the stem of a diseased bitter gourd plant collected in Hunan Province, China. Strain HuN8 displayed a similar colony morphology to strain SD-1 (virus free) on PDA medium (Figure 1A) and induced slight leaf yellowing, while the virus-free strain SD-1 induced obvious leaf yellowing (Figure 1B). The dsRNA was extracted from strain HuN8 and treated with DNase I to digest the genomic DNA. We found that two segments, the L-segment and the S-segment, were harbored in strain HuN8 (Figure 1C).

### Molecular Characterization of FoOuLV1

**FIGURE 2:**
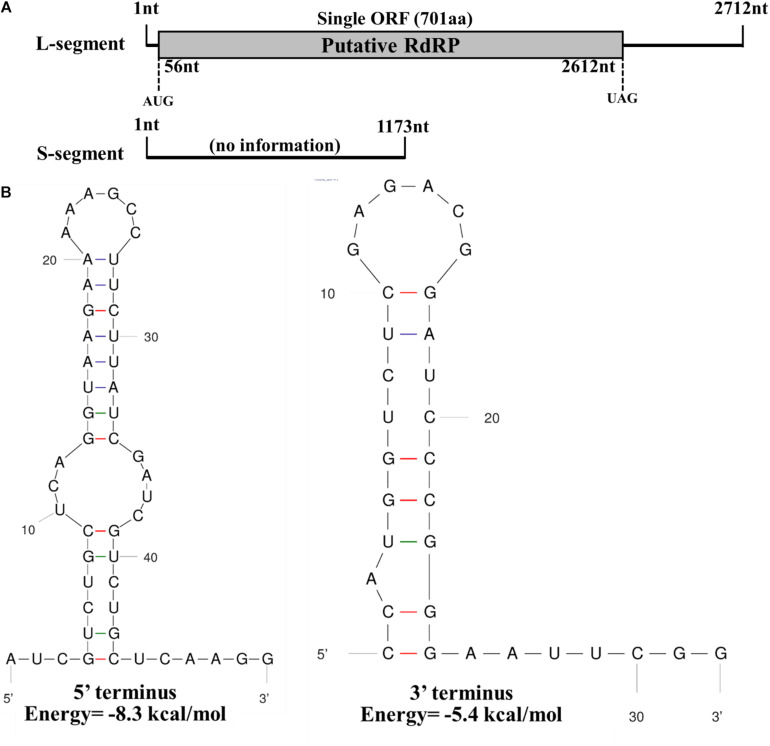
Genomic organization and terminal structure of *Fusarium oxysporum* ourmia-like virus 1 (FoOuLV1). **(A)** Schematic representation of the putative genomic organization of FoOuLV1. The open reading frame (ORF) shown as a gray box (56–2,162 nt) was putatively encoding the RNA-dependent-RNA polymerase (RdRp) domain. **(B)** Potential secondary structures of the 5′- (left) and 3′-termini (right) of the L fragment of FoOuLV1.

The full-length sequence of the L-segment (GenBank Accession No. MT551010) in FoOuLV1 was 2,712 nt with a GC content of 55.5%. The genome contained only one large ORF that initiated at position 56 and terminated at position 2,612 based on the universal or mitochondrial genetic codes, potentially encoding 701 amino acid residues with a calculated molecular mass of 78.9 kDa from an AUG triplet to a UAG triplet (Figure 2A). Using the Mfold RNA structure software, the complex secondary structures of the L-segment of FoOuLV1 were predicted. The results indicated that the first 50 nt at the 5′ terminus were folded into two stable stem–loop structures, while the last 32 nt at the 3′ terminus formed a stable stem–loop structure (Figure 2B). Stem–loop structures are typical features of members of *Narnaviridae* (including mitovirus) and *Ourmiavirus*.

**FIGURE 3:**
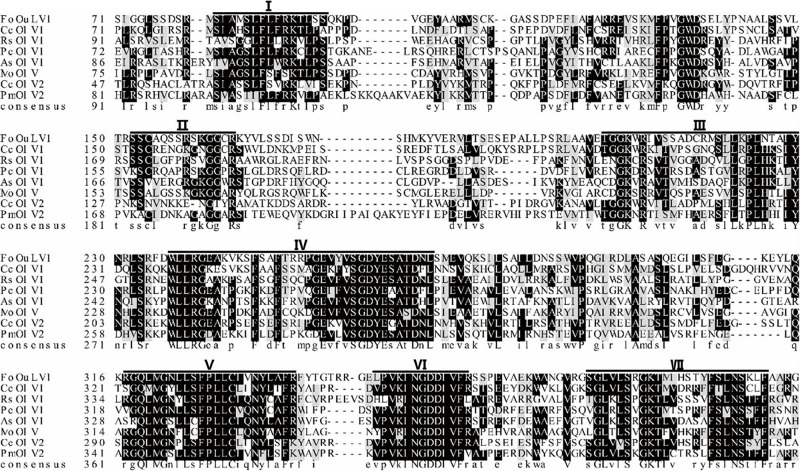
Multiple alignment of the amino acid sequences of RNA-dependent RNA polymerase (RdRp) proteins encoded by FoOuLV1 and other selected ourmia-like viruses. Horizontal lines above the alignment indicate seven motifs (Roman numerals I to VII). Abbreviations: CcOLV1, *Cladosporium cladosporioides* ourmia-like virus 1; RsOLV1, *Rhizoctonia solani* ourmia-like virus 1; PcOLV1, *Penicillium citrinum* ourmia-like virus 1; AsOLV1, *Acremonium sclerotigenum* ourmia-like virus 1; MoOLV, Magnaporthe oryzae ourmia-like virus; CcOLV2, *Cladosporium cladosporioides* ourmia-like virus 1; and PmOLV2, *Phaeoacremonium minimum* ourmia-like virus 2.

Using the homology search on BLASTP, the ORF encoded a protein with one conserved domain, which was closely related to RdRps of Penicillium citrinum ourmia-like virus 1, Cladosporium cladosporioides ourmia-like virus 2, and Phaeoacremonium minimum ourmia-like virus 2 (Table 1). Furthermore, a conserved domain database (CDD) search and multiple protein alignment suggested that the predicted RdRp domain contained seven conserved motifs (Figure 3).

**TABLE 1:** Identifications of the RdRp of FoOuLV1 and those of ourmia-like mycoviruses.

Taxon	Virus name	Accession	Query cover (%)	Identity (%)	*E* value
Magoulivirus	*Penicillium citrinum* ourmia-like virus 1	AYP71797.1	66	38.87	3.00E–83
	*Cladosporium cladosporioides* ourmia-like virus 2	QDB75008.1	77	35.93	8.00E–81
	*Phaeoacremonium minimum* ourmia-like virus 2	QDB75007.1	77	35.55	2.00E–77
Scleroulivirus	Soybean leaf-associated ourmiavirus 1	YP009666497.1	44	33.02	3.00E–34
	*Sclerotinia sclerotiorum* ourmia-like virus 1	ALD89138.1	43	32.15	3.00E–33
	*Pyricularia oryzae* ourmia-like virus 3	BBF90578.1	41	34.85	3.00E–31
Botoulivirus	*Sclerotinia sclerotiorum* ourmia-like virus 2	ALD89139.1	33	29.67	9.00E–22
	*Epicoccum nigrum* ourmia-like virus 1	QDB75003.1	28	31.73	2.00E–28
	Entoleuca ourmia-like virus 1	AVD68674.2	32	30.92	2.00E–24
Penoulivirus	*Penicillium sumatrense* ourmia-like virus 1	QDB75000.1	39	29.74	2.00E–21
	*Sclerotinia sclerotiorum* ourmia-like virus 4	QHG11400.1	40	27.70	4.00E–16
	*Aspergillus neoniger* ourmia-like virus 1	AZT88620.1	30	31.36	2.00E–16
Ourmiavirus	Epirus cherry virus	ACF16357.1	42	26.22	8.00E–20
	Ourmia melon virus	YP002019757.1	22	33.90	5.00E–21
	Cassava virus C	YP003104770.1	22	32.00%	4.00E–16

The full-length sequence of the S-segment (GenBank Accession No. MT551011) associated with FoOuLV1 was composed of 1,173 nt with a GC content of 43.8%, and the segment contained no ORFs (Figure 2A). There was no significant similar information from NCBI database using BLASTX.

### Phylogenetic Analysis of FoOuLV1

**FIGURE 4:**
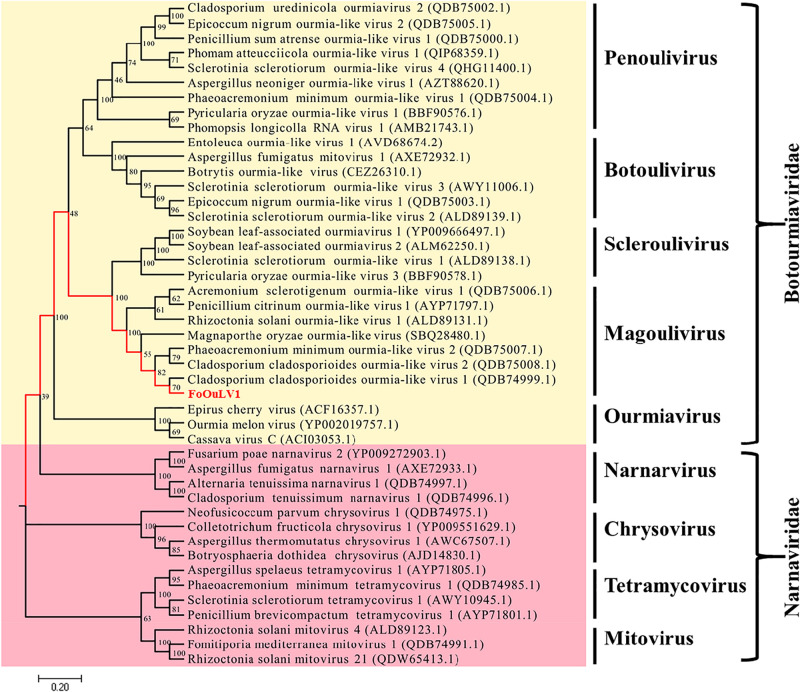
Maximum likelihood phylogenetic tree based on the RdRps of FoOuLV1 and other related viruses. The phylogenetic analysis was generated using the MEGA7 software with 1,000 bootstrap replicates. The scale bar represents a genetic distance of 0.2.

To examine the phylogenetic relationship between FoOuLV1 and other mycoviruses, the phylogenetic tree was constructed using a maximum likelihood method based on the RdRp amino acid sequences of FoOuLV1 and other related viruses from *Narnaviridae* (including *Narnarvirus*, *Chrysovirus*, *Tetramycovirus*, and *Mitovirus*) and *Botourmiaviridae* (including *Penoulivirus*, *Botoulivirus*, *Scleroulivirus*, *Magoulivirus*, and *Ourmiavirus*). The results indicated that FoOuLV1 was closely related to the genus *Magoulivirus* and clustered with viruses such as Cladosporium cladosporioides ourmia-like virus, Phaeoacremonium minimum ourmia-like virus 2, and Magnaporthe oryzae ourmia-like virus. Thus, FoOuLV1 was determined to be a new member of *Magoulivirus* within the family *Botourmiaviridae* (Figure 4).

### Influence of FoOuLV1 to the Virulence of *FoM*

Pathogenicity detection of FoOuLV1-containing strain HuN8 and virus-free strain SD-1 on the detached bitter gourd leaves showed that FoOuLV1 reduced the virulence of the *FoM* strain as described above (Figure 1B). Fusarium wilt caused by *F. oxysporum* is a systemic infection disease characterized by a typical symptom of whole plant wilting. In order to further determine whether the presence of FoOuLV1 reduced the virulence of *FoM* strain in terms of infecting living bitter gourd plants, the *FoM* strains HuN8 and SD-1 were inoculated onto bitter gourd seedlings at the stage of two or three leaves. Pathogenicity data were investigated before, during, and after the presentation of typical wilt symptoms.

**FIGURE 5:**
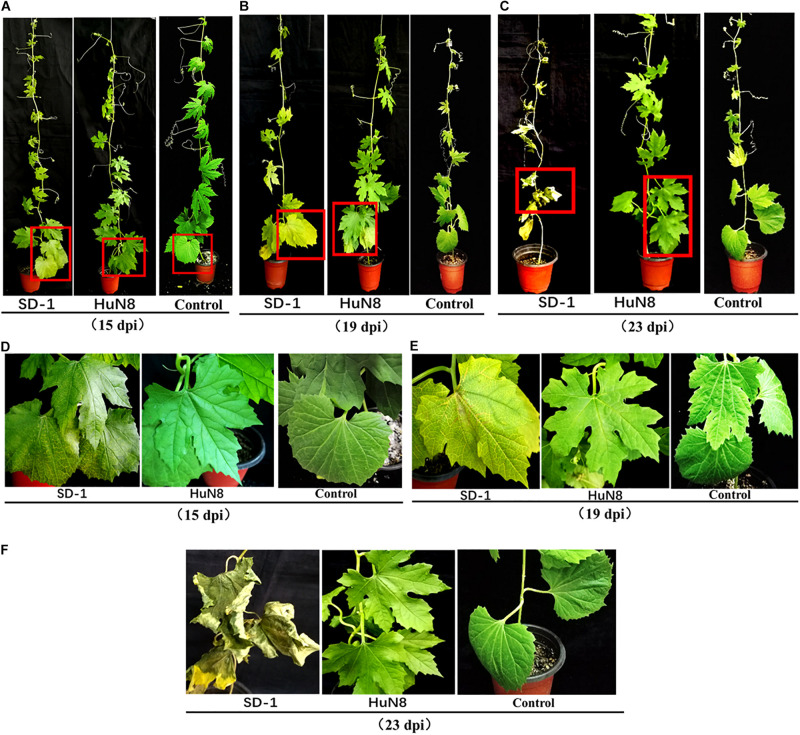
Pathogenicity detection of strains HuN8 and SD-1 in living bitter gourd seedlings. **(A–C)** Symptoms at the early (15 dpi), middle (19 dpi), and later (23 dpi) stages. **(D–F)** The enlarged images of the red boxes in **(A**–**C)**, respectively.

At the early stage (15 dpi), the basal leaves of bitter gourd inoculated with SD-1 strain showed the symptoms of vein fading and yellowing on leaves, while the leaves of bitter gourd inoculated with HuN8 strain showed no symptoms (Figures 5A,D). At the middle stage (19 dpi), most of the leaves of bitter gourd inoculated with SD-1 strain had shown the typical symptoms of Fusarium wilt (leaf vein fading and yellowing), while those leaves inoculated with HuN8 strain still showed no symptoms (Figures 5B,E). At the later stage (23 dpi), the whole bitter gourd plant inoculated with SD-1 strain exhibited dryness and wilting, while the bitter gourd inoculated with HuN8 strain remained no symptoms during the entire observation period (Figures 5C,F).

### Horizontal Transmission of FoOuLV1 Among FoM Strains

**FIGURE 6:**
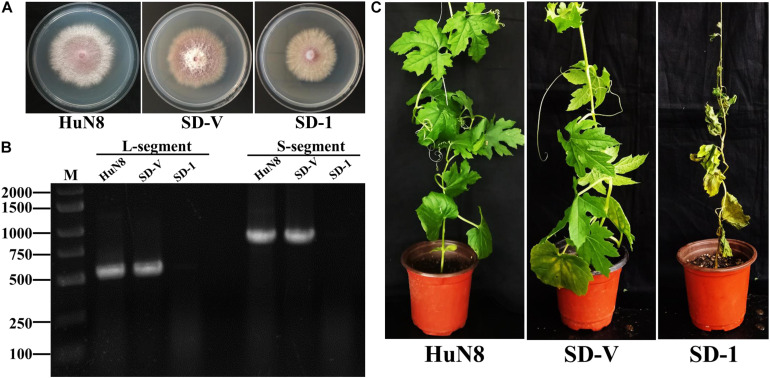
FoOuLV1 infectivity and pathogenicity in *FoM* strains. **(A)** Colony morphology of the HuN8 strain, the virulent strain SD-1, and the derivative strain SD-V (cultured on PDA for 2 days at 28°C). **(B)** Detection of the L- and S-segments by RT-PCR. M, DNA marker. **(C)** Pathogenicity detection in a living bitter gourd seedling.

The dual-culture technique was used to determine whether the hypovirulence traits and RNA elements of FoOuLV1 could be transmitted horizontally. The results indicated that the L- and S-elements were both successfully transmitted from the hypovirulent strain HuN8 to the mycovirus-free strain SD-1Hyg^*R*^, yielding the derivative strain SD-V. The colony morphology of strain SD-V was similar to that of the strain SD-1 (Figure 6A). The presence of L- and S-segments was confirmed in strain SD-V by dsRNA extraction (Supplementary Figure 4A) and RT-PCR with specific primers (Figure 6B). The hypovirulence traits were also successfully transmitted, as indicated by the pathogenicity detection results: the whole bitter gourd plants inoculated with strain SD-1 exhibited dryness and wilting, while plants inoculated with strain SD-V showed no symptoms, appearing in the same way as the strain HuN8 (Figure 6C).

### Horizontal Transmission of FoOuLV1 Between Different Special Form Strains of *F. oxysporum*

**FIGURE 7:**
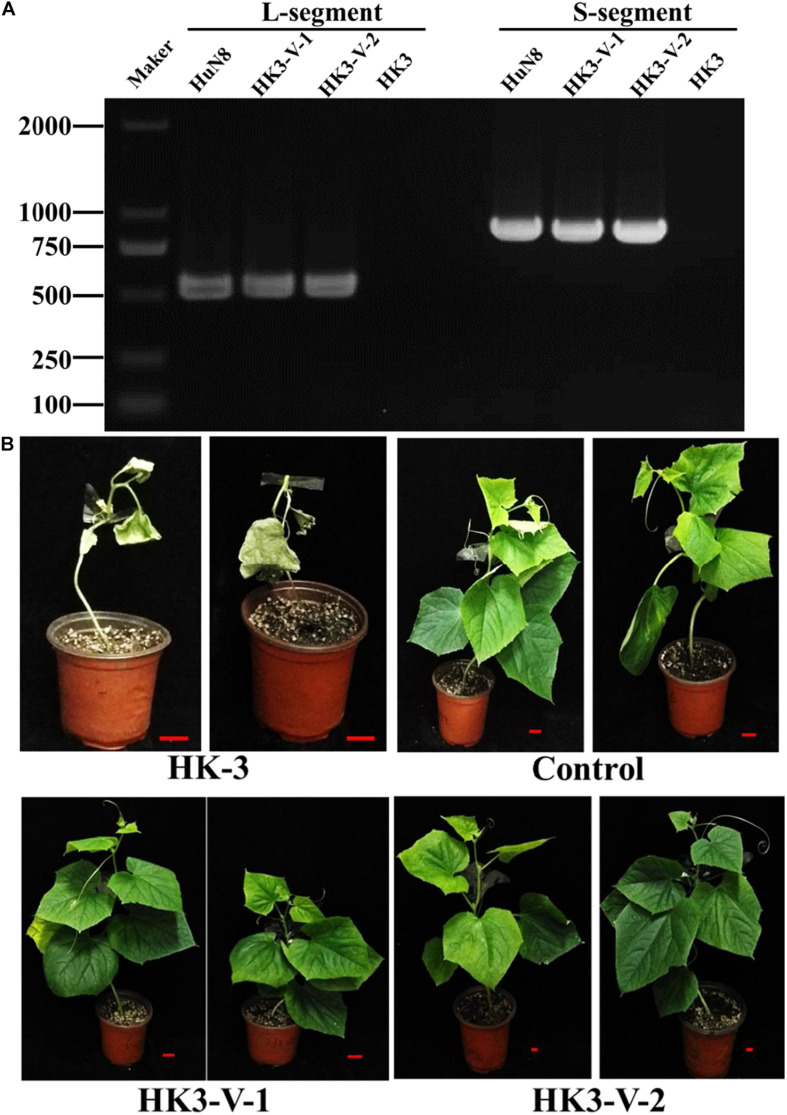
FoOuLV1 infectivity and pathogenicity in FoC strains. **(A)** Detection of L- and S-segments by RT-PCR. **(B)** Pathogenicity detection in living cucumber seedlings. HuN8 is the FoM strain (with FoOuLV1), HK3 is the mycovirus-free FoC strain, HK3-V-1, and HK3-V-2 are the FoC derivative strains obtained from the dual-culture technique with strains HuN8 and HK3.

The specialization of *F. oxysporum* is closely related to its pathogenicity. In order to determine whether the hypovirulent traits could be transmitted from *FoM* strain to *FoC* strain, dual culture was performed. The results showed the L- and S-segments were successfully transmitted from *FoM* strain HuN8 to virus-free *FoC* strain HK3Hyg^*R*^, yielding the derivative strains HK3-V-1 and HK3-V-2. The L- and S-segments were detected by dsRNA extraction (Supplementary Figure 4B) and RT-PCR (Figure 7A). The hypovirulent traits were also successfully transmitted: whole cucumber plants inoculated with the strain HK3 exhibited dryness and wilting, while plants inoculated with strains HK3-V-1 and HK3-V-2 showed no symptoms (Figure 7B).

### Biological Control Effects of FoOuLV1 Against Fusarium Wilt in Bitter Gourd

To verify the biological control effect of FoOuLV1 against Fusarium wilt in bitter gourd plants, field experiment was conducted from 21 May to 16 July 2019 in two fields. The results are shown in Table 2. According to the first results obtained from Field 1 on 25th June, the disease incidence of bitter gourd Fusarium wilt in the control group was 29.03% and the disease index was 18.28. In contrast, in the mycovirus FoOuLV1-treated group, the disease incidence was only 7.69%, the disease index was only 3.42, and the control effect reached 81.29%. A similar result was observed in Field 2, where the control effect reached 78.92%. The second results collected on 16th July showed a similar control effect (79.76% in Field 1 and 88.10% in Field 2; Table 1).

**TABLE 2:** Control effects of FoOuLV1 against *Fusarium* wilt in bitter gourd in field.

	Numbers of each level									
								Disease		
	Level 0	Level 1	Level 3	Level 5	Level 7	Level 9	Total number	incidence (%)	Disease index	Control effect (%)
**Field 1**										
**First statistics (June 25)**										
Control	22	3	0	0	3	3	31	29.03	18.28	81.29
SD-V	24	0	1	1	0	0	26	7.69	3.42	
**Second statistics (July 16)**										
Control	11	5	3	1	6	5	31	64.52	37.99	79.76
SD-V	22	1	1	1	0	1	26	15.38	7.69	
**Field 2**										
**First statistics (June 25)**										
Control	31	0	2	3	1	0	37	16.22	8.4	78.92
SD-V	23	1	1	0	0	0	25	8.00	1.77	
**Second statistics (July 16)**										
Control	20	2	3	6	4	2	37	45.95	26.13	88.10
SD-V	22	1	2	0	0	0	25	12.00	3.11	

*Note: The disease incidence=The⁢total⁢numbers⁢of⁢level⁢ 1⁢to⁢level⁢ 9Total⁢numbersof⁢level⁢ 0⁢to⁢level⁢ 9×100%. The disease index=Sum(thevalueofeachlevel×thenumbersonthislevel)fromlevel 0tolevel 9)Total⁢numbers×the⁢value⁢of⁢the⁢highest⁢level. The control effect$=\frac{\mathrm{The}\,\mathrm{disease}\,\mathrm{index}\,\mathrm{of}\,\mathrm{Control}-\mathrm{the}\,\mathrm{disease}\,\mathrm{index}\,\mathrm{of}\,\mathrm{SD}-\text{V}}{\mathrm{The}\,\mathrm{disease}\,\mathrm{index}\,\mathrm{of}\,\mathrm{Control}}\times 100\%$.*

## Discussion

### Genomic Differences Between FoOuLV1 and Other Ourmia-Like Viruses

Recently, an increasing number of ourmia-like viruses have been reported in diverse fungi, including two ourmia-like viruses from *Magnaporthe oryzae* (Illana et al., 2017; Li C.X. et al., 2019), three ourmia viruses from *Pyricularia oryzae* (Ohkita et al., 2019), two ourmia viruses from *Sclerotinia sclerotiorum* (Marzano et al., 2016; Wang et al., 2020), and other ourmia viruses from *Botrytis* (Donaire et al., 2016), *Phomopsis longicolla* (Hrabáková et al., 2017), and *Phoma matteucciicola* (Zhou et al., 2020). All of them are phylogenetically related to those in the newly established family *Botourmiaviridae*, which currently comprised five genera, according to current taxonomic information from ICTV and research report. Members of the genus *Ourmiavirus* are found only in plant-infecting viruses and usually harbor three RNA segments encoding RdRp, MP, and CP protein. Viruses in the genera *Botoulivirus*, *Penoulivirus*, and *Magoulivirus* are only isolated from fungi, while *Scleroulivirus* viruses are isolated from both fungi and plants. The mycoviruses in these four genera contain only one RNA segment encoding RdRp (Crivelli et al., 2011; Wang et al., 2020).

In this study, we described a novel ourmia-like mycovirus FoOuLV1, which is identified in *F. oxysporum* for the first time and a new member of the genus *Magoulivirus* within the family *Botourmiaviridae*. The genome of FoOuLV1 only has one ORF encoding RdRp in the L-segment similar to the other ourmia-like mycoviruses (Guo et al., 2019; Wang et al., 2020; Zhou et al., 2020). In addition, it contains a shorter segment named S-segment, which has no blast information. In this regard, the FoOuLV1 is significantly different from other ourmia-like mycoviruses.

### The FoOuLV1 Is Unique Compared With Other Ourmia-Like Mycoviruses

Some mycoviruses in pathogenic fungi can cause a decline in the pathogenicity of the host fungi. This is known as mycovirus-mediated hypovirulence and has attracted research attention, owing to its importance in controlling fungal diseases (Nuss, 2005; Suzuki et al., 2018). To date, many families of mycoviruses have been identified as being associated with fungal hypovirulence, including the following: *Hypoviridae* (e.g., CHV1), *Megabirnaviridae* [e.g., Rosellinia necatrix megabirnavirus 1 (RnMBV1)], *Reoviridae* [e.g., Rosellinia necatrix mycoreovirus 3 (RnMYRV-3)], and *Narnaviridae* [e.g., Sclerotinia sclerotiorum mitovirus 1 (SsMV1)] (Hillman et al., 1990; Kanematsu et al., 2004; Sasaki et al., 2007; Chiba et al., 2009; Xu et al., 2015). In addition, some unclassified hypoviruses (such as SsHADV-1 and Sclerotinia sclerotiorum hypovirus 1) have been recognized as inducing hypovirulence (Yu et al., 2010; Xie et al., 2011).

A large number of ourmia-like viruses have been reported in diverse fungi, but none of them exhibit hypovirulence. As reported in this study, FoOuLV1 is the first ourmia-like mycovirus to be reported that exhibits hypovirulence. The S-segment associated with FoOuLV1 is unique among ourmia-like mycoviruses. A previous study showed that SsHV1 and its satellite-like RNA were able to co-infect the hypovirulent strain SZ-150, with the satellite-like RNA in conferring hypovirulence on *S. sclerotiorum* (Xie et al., 2011). Therefore, S-segment associated with FoOuLV1 may play an important role in its hypovirulence.

### The Transmission of FoOuLV1 Is Important for *F. oxysporum*

Mycoviruses are generally transmitted by hyphal anastomosis and during sporogenesis, with the hyphal anastomosis occurring naturally between individuals belonging to the same or closely related vegetative compatibility groups (Lee et al., 2014). Furthermore, transmission via protoplast fusion has been reported in many mycoviruses (van Diepeningen et al., 1998; Kanematsu et al., 2010). The efficient transmission observed among isolates under natural conditions is considered to be an important condition for the successful application of mycoviruses to the control of plant fungal disease.

Fusarium wilt caused by *F. oxysporum* affects a large number of economically important crops. The pathogenic species contain a diversity of host-plant–specific forms (Torres-Trenas and Pérez-Artés, 2020), with different formae speciales potentially existing together in the soil concurrently. The hypovirulent traits of FoOuLV1 reported here can be transmitted horizontally from *FoM* strains to virus-free *FoM* strains and to *FoC* strains. This indicates that FoOuLV1 could be potentially used to control Fusarium wilt in various crops.

### Potential Use of FoOuLV1

A large number of hypovirulent mycoviruses have been identified in previous studies, and some have been explored as potential biocontrol agents against fungal diseases. CHV1 has successfully been used to control chestnut blight in Europe (Anagnostakis, 1982), while SsHADV1 was used to control the disease caused by *S. sclerotiorum* (Yu et al., 2013). Except for these two mycoviruses, attempts to control diseases in field using mycoviruses are rare.

Many mycoviruses have been identified from the genus *Fusarium* (Cho et al., 2013); but, to our knowledge, only three mycoviruses have been identified in *F. oxysporum* (FoCV1, FodV1, and FodMV1). Furthermore, among these, only FodV1 induces hypovirulence (Sharzei et al., 2007; Lemus-Minor et al., 2015; Torres-Trenas and Pérez-Artés, 2020). In this study, FoOuLV1 was not only able to induce hypovirulence in *F. oxysporum* but also exhibited significant biological control effects against bitter gourd Fusarium wilt in the pot and field experiments. This suggests that FoOuLV1 has a potential to be used in the future.

## Conclusion

In this study, we characterized a novel mycovirus (FoOuLV1) related to members of the *Magoulivirus* genera from a phytopathogenic fungus, *F. oxysporum* f. sp. *momordicae*. Although some ourmia-like mycoviruses have been identified in diverse fungi, this is the first report of a mycovirus in *F. oxysporum* f. sp. *momordicae* and the first report of an ourmia-like mycovirus in *F. oxysporum*. Furthermore, FoOuLV1 is the first ourmia-like mycovirus harboring an associated RNA segment and possessing a hypovirulence-inducing trait. Therefore, this virus provides a valuable experimental system to study the interaction of ourmia-like mycovirus and its fungal host. In addition, FoOuLV1 is also a potential biocontrol agent that could be further studied to control Fusarium wilt.

## Data Availability Statement

The sequence file of FoOuLV1 is available from the NCBI, GenBank Accession Nos. MT551010 and MT551011.

## Author Contributions

YiZ designed the research. YuZ, YS, ML, XW, HX, and YiZ performed the experimental work. YiZ, JX, and CW analyzed the data and wrote the manuscript. All authors contributed to the article and approved the submitted version.

## Conflict of Interest

The authors declare that the research was conducted in the absence of any commercial or financial relationships that could be construed as a potential conflict of interest.

## Footnotes

1 http://www.ncbi.nlm.nih.gov

2 http://www.genome.jp/tools/motif/
