# Supplementary material for: A Novel Ourmia-Like Mycovirus Confers Hypovirulence-Associated Traits on Fusarium oxysporum
Source: Front Microbiol. 2020 Dec 9;11:569869. doi: 10.3389/fmicb.2020.569869 (PMC7756082; doi:10.3389/fmicb.2020.569869)
Supplement: Supplementary file 1 [file Data_Sheet_1.zip › Fig S1.DOCX]

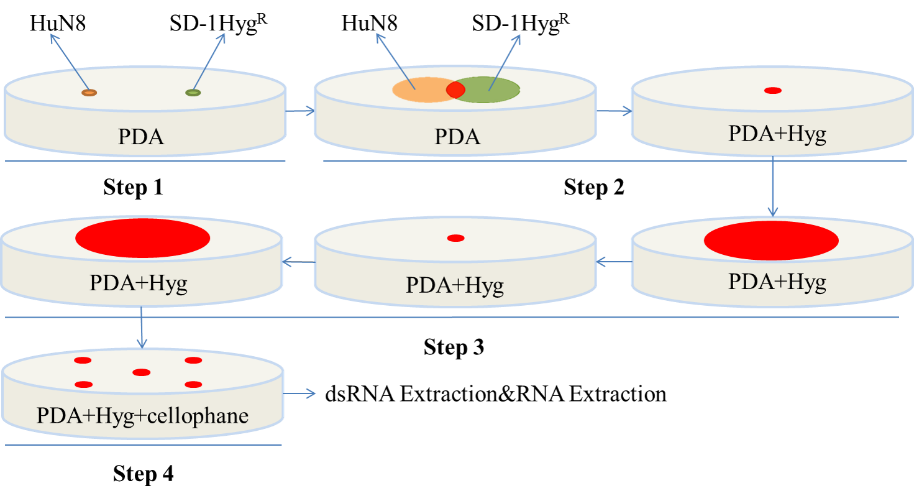


**Figure S1.** Schematic diagram for co-culturing performed. There were four steps in the co-culturing performed method. **Step 1:** the strains HuN8 and SD-1HygR (or HK3HygR) were cultured in the same PDA plate growing for 4–5 days, and the distance between strains HuN8 and the SD-1HygR (or HK3HygR) was about 10-15mm. **Step 2:** the strains HuN8 and SD-1HygR (or HK3HygR) were grown until the colony of HuN8 and the SD-1HygR (or HK3HygR) covered each other. Then the common colony (**the rad part**) of HuN8 and the SD-1HygR (or HK3HygR) were transferred to new PDA plates with hygromycin (50 mg/mL). **Step 3:** the colony which could grow on PDA plates with hygromycin were transferred to new PDA plates with hygromycin again for purifying the derivative strains. **Step 4:** the derivative strains were cultured on the cellophane membrane overlying a PDA plate with hygromycin to obtained hyphae for dsRNA extraction or RT-PCR detection.
