# Supplementary material for: A Novel Ourmia-Like Mycovirus Confers Hypovirulence-Associated Traits on Fusarium oxysporum
Source: Front Microbiol. 2020 Dec 9;11:569869. doi: 10.3389/fmicb.2020.569869 (PMC7756082; doi:10.3389/fmicb.2020.569869)
Supplement: Supplementary file 1 [file Data_Sheet_1.zip › Fig S2.DOCX]

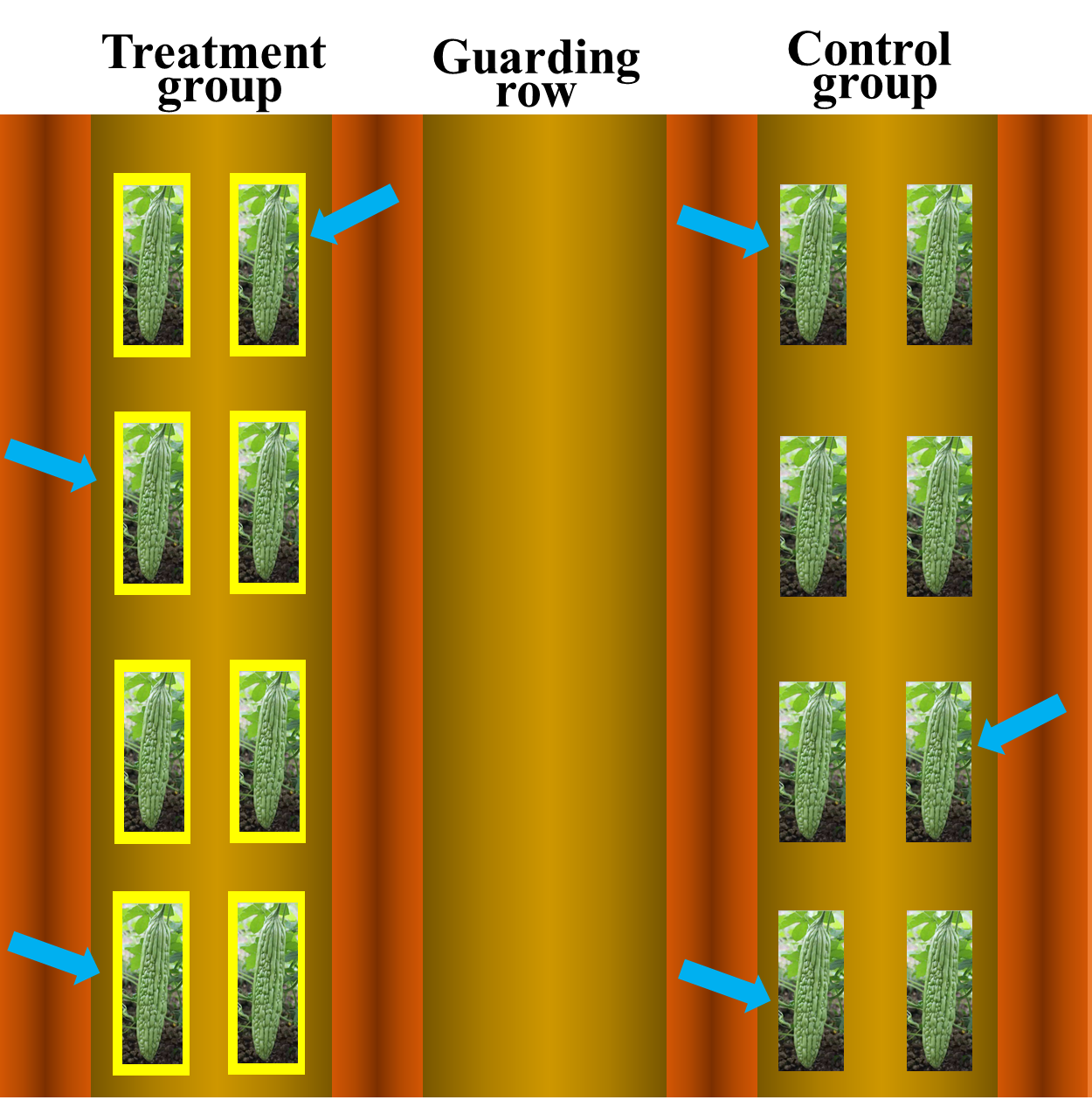


**Figure S2.** The schematic representation of field experiment distribution. The treatment and control groups are grew bitter gourd, the guarding row is growing nothing. The treatment group were inoculated with 10 mL spores (10^7^ mL^-1^) of the SD-V strain, the control group was inoculated with water. The blue arrows represent random sampling points.
