# Supplementary material for: A Novel Ourmia-Like Mycovirus Confers Hypovirulence-Associated Traits on Fusarium oxysporum
Source: Front Microbiol. 2020 Dec 9;11:569869. doi: 10.3389/fmicb.2020.569869 (PMC7756082; doi:10.3389/fmicb.2020.569869)
Supplement: Supplementary file 1 [file Data_Sheet_1.zip › Fig S3.DOCX]

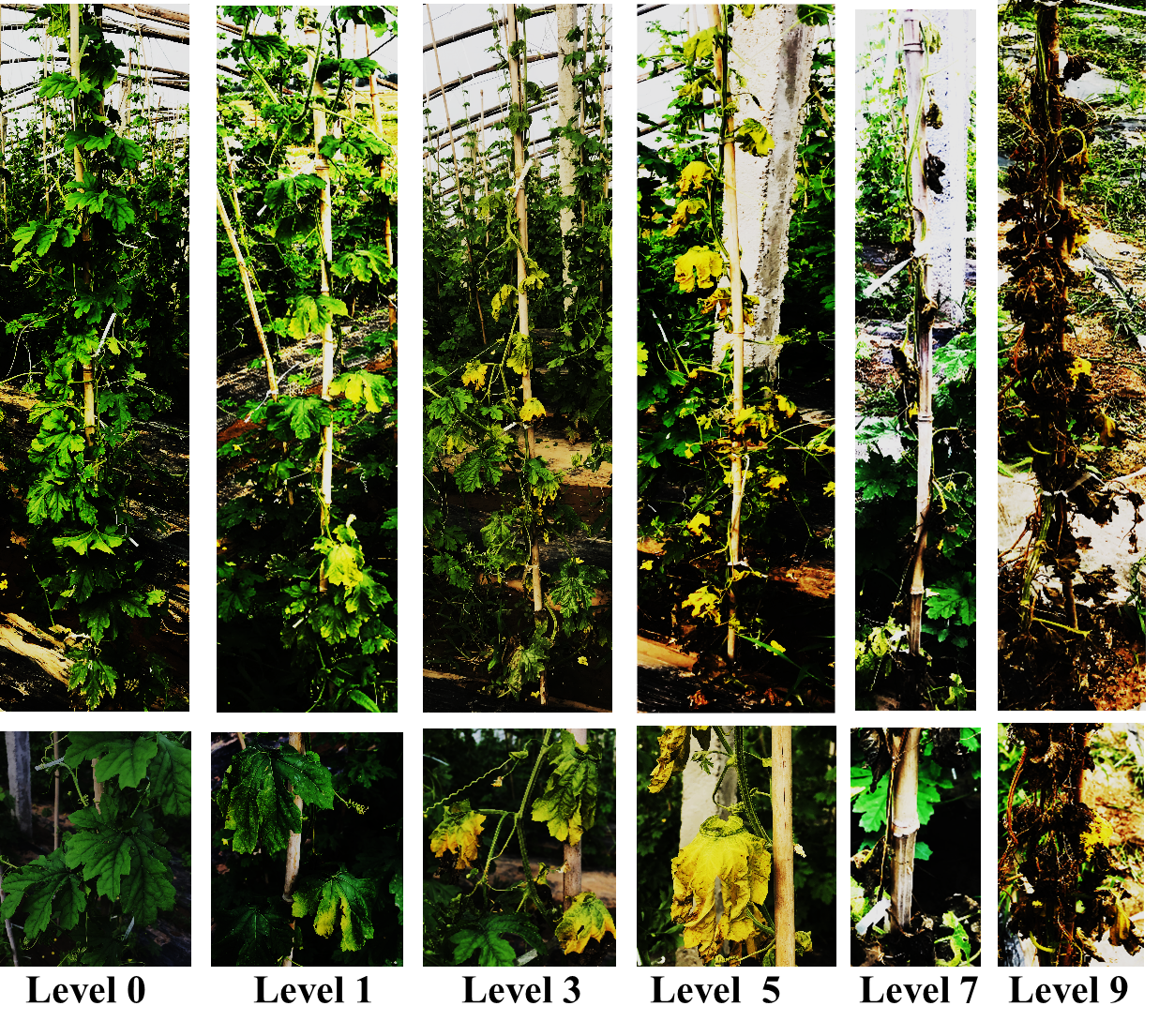


**Figure S3.** The different levels symptoms of bitter gourd fusarium wilt. level 0, no symptoms; level 1, <30% of leaves show leaf veins with yellowing; level 3, 30%–70% of leaves show leaf veins with yellowing; level 5, leaf veins on the whole plant are yellowed, but the growth and development of the plant are not affected; level 7, the whole plant is yellowed and wilted, and the vascular bundle has turned brown and stopped growing and developing; level 9, the plant is died.
