# Supplementary material for: A Novel Ourmia-Like Mycovirus Confers Hypovirulence-Associated Traits on Fusarium oxysporum
Source: Front Microbiol. 2020 Dec 9;11:569869. doi: 10.3389/fmicb.2020.569869 (PMC7756082; doi:10.3389/fmicb.2020.569869)
Supplement: Supplementary file 1 [file Data_Sheet_1.zip › Fig S4.DOCX]

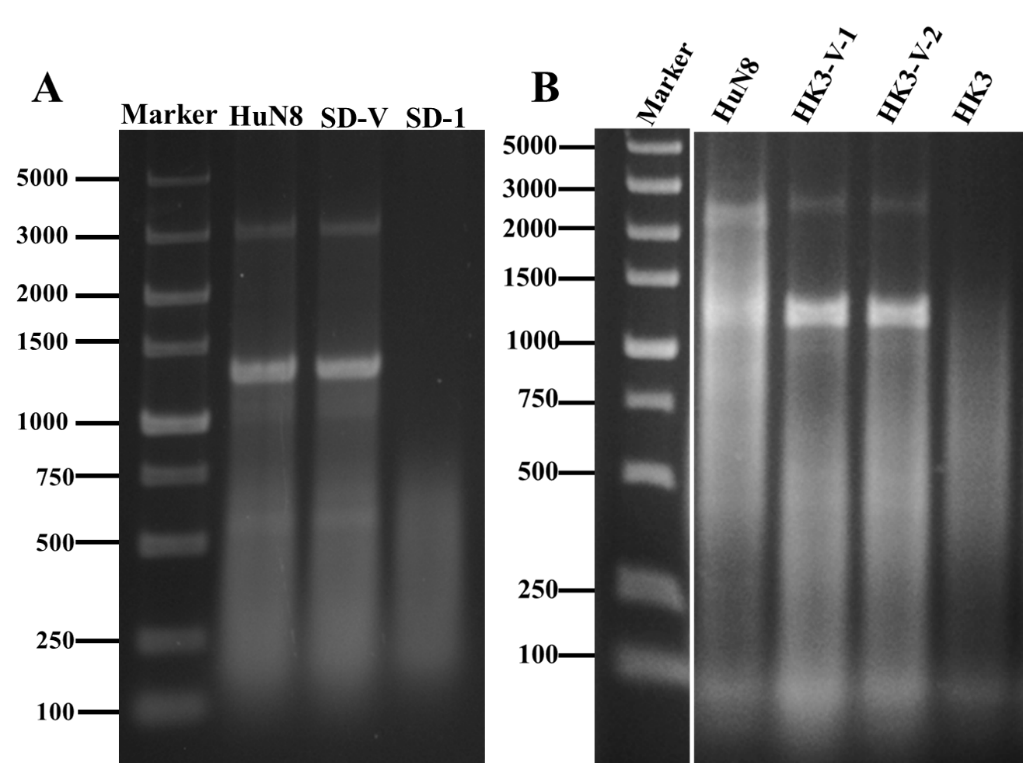


**Figure S4.** Agarose gel electrophoresis analysis of the dsRNA extracted from derivative strains. **(A)** The dsRNA extracted from *FoM* derivative strains. **(B)** The dsRNA extracted from *FoC* derivative strains.
