# Supplementary material for: A Novel Ourmia-Like Mycovirus Confers Hypovirulence-Associated Traits on Fusarium oxysporum
Source: Front Microbiol. 2020 Dec 9;11:569869. doi: 10.3389/fmicb.2020.569869 (PMC7756082; doi:10.3389/fmicb.2020.569869)
Supplement: Supplementary file 1 [file Data_Sheet_1.zip › Tabe S1.DOCX]

**Table S1.** A list of primers used in this study.

| Primer Name | Oligonucleotide sequence (5′- 3′) | Usage |
| --- | --- | --- |
| ITS1 | TCCGTAGGTGAACCTGCGG | For Ribosomal RNA ITS region of *Fusarium oxysporum* f.sp*. momordicae* |
| ITS4 | TCCTCCGCTTATTGATATGC |  |
| fp7318 | AGGTGCAGCGTTTTTAGGT | Specific primers of *Fusarium oxysporum* f.sp. *momordicae* |
| fp7335 | GAGGGCTGGTTGAGAACTA |  |
| RACE3RT | CGATCGATCATGATGCAATGCNNNNNN | For initial sequence cloning |
| RACE3 | CGATCGATCATGATGCAATGC |  |
| pC3-T7loop | GGATCCCGGGAATTCGGTAA  TACGACTCACTATATTTTTATA  GTGAGTCGTATTA | For terminal sequence cloning |
| pC2 | CCGAATTCCCGGGATCC |  |
| FV-L-S | ATCAGGCGATAATGGCGTGTA | Specific primers of L-segment of FoOuLV1 |
| FV-L-A | CGGCGTAGGTTCGTGTTGTT |  |
| FV-S-S | AGCTCACCTGCCACCTATCCT | Specific primers of S-segment of FoOuLV1 |
| FV-S-A | GACAATGCCGCTAGTCGAAGT |  |
